# An analysis of data sources and study registries used in systematic reviews

**DOI:** 10.1111/wvn.12614

**Published:** 2022-11-15

**Authors:** Jan M. Nick, Nancy L. Sarpy

**Affiliations:** ^1^ Loma Linda University ‐ School of Nursing Loma Linda California USA

**Keywords:** bibliographic analysis, data sources, database, informatics, language bias, pre‐appraised literature, PRISMA‐S, reproducibility, search strategies, systematic reviews

## Abstract

**Background:**

Reporting standards for data sources in systematic reviews (SRs) have been developed, yet research shows varying compliance in the methods section. When this happens, replication of search results is difficult and creates ambiguous and biased data sources.

**Aims:**

This study captured author practices in choosing English and non‐English‐language databases, listing all the databases searched, and incorporating study registries as part of the search strategy.

**Methods:**

Using an analytic, cross‐sectional, study design, volunteer data collectors (*n* = 107) searched one of two assigned English language platforms for SRs on specified health conditions. All the data sources found in the methods section of each SR were documented and analyzed for patterns using bibliographic techniques.

**Results:**

The final sample size of the SRs reviewed was *N* = 199. The mean number of data sources seen in the SRs was 3.9 (SD 2), with a range of 1–10. Eighteen records (9%) used a single data source to conduct the SRs. Four leading language platforms were seen in the SRs: English (100% of occurrences), up to 8% used Chinese data sources, and 4% included Spanish or Portuguese. The four most frequently used data sources were: (1) Medline (98%), (2) Embase (65%), (3) Cochrane Library (56%), and (4) Web of Science (33%). The percentage of SRs listing study registries was 30%.

**Linking Evidence to Action:**

Strategies to reduce bias and increase the rigor and reliability of SRs include comprehensive search practices by exploring non‐English‐language databases, using multiple data sources, and searching study registries. By following PRISMA‐S guidelines to report data sources correctly, reproducibility can be accomplished.

## BACKGROUND

Systematic reviews (SRs) are the cornerstone of evidence‐based practice (EBP), as they provide a synthesis of sound primary research studies found in a variety of published and unpublished sources. Authors refer to SRs as the gold standard (Aromataris & Pearson, 2014), or the highest form of evidence (Harwood et al., 2019), and are currently the most frequently cited type of clinical research (Djulbegovic & Guyatt, 2017). Because of the clinical value and weight placed on this evidence, rigorous search methods are essential to obtain high‐quality SRs. Care must also be taken to correctly document data sources. To this end, the Preferred Reporting Items for Systematic Reviews and Meta‐Analyses‐Searching extension (PRISMA‐S) sets clear standards for the inclusion of key items for authors and publishers to follow prior to publication of the SR (Salvador‐Oliván et al., 2019). The standards include 16 items divided into four sections: (1) information sources and methods, (2) search strategies, (3) peer review, and (4) managing records (Rethlefsen et al., 2021). However, research has recently shown that 90% of SRs lack comprehensive search strategies and specificity in data sources reporting details (de Kock et al., 2020). When specificity in search parameters and reporting details are lacking, replication and obtaining similar results are difficult (Biocic et al., 2019; Golder et al., 2019).

SRs first appeared in the healthcare literature in 1991, and for several years, only a few were published (Greenhalgh, 1994; O'Day et al., 1993; Pinson et al., 1991; Vander Stichele et al., 1995). Then in 1995, a global network of researchers established the Cochrane Database of Systematic Reviews (CDSRs), and the production of SRs has increased steadily since then (Aldington & Eccleston, 2019). During this time of maturation in synthesis science, health science librarians played a significant supporting role in the development of search and retrieval standards for SRs (Boden et al., 2018), as well as increased the overall quality of the SRs (Rethlefsen et al., 2015). To this end, including health science librarians on the team is now an expectation when conducting any type of pre‐appraised research.

The importance of a well‐constructed SR cannot be underscored enough as it has been shown to decrease the incidence of publication (Faggion et al., 2016) and language bias (Amano et al., 2016; Cohen et al., 2015; Grzybowski & Kanclerz, 2019; Konno et al., 2020; Rasmussen & Montgomery, 2018; Rethlefsen et al., 2015; Stern & Kleijnen, 2020) and aid in the identification of reporting biases (Bethel & Rogers, 2014; Mayo‐Wilson et al., 2018). Confirmation bias, choosing only what supports prior beliefs or attitudes, has the highest chance of occurring during the search process (Vedejová & Čavojová, 2022). Fortunately, the duplicate yet independent decision‐making by multiple persons in each step of the systematic review process diminishes this bias. Unfortunately, it cannot be eliminated completely. Results from recent bibliometric research showed almost 10% of SRs showed this type of bias when searching as authors looked only for outcomes they expected to find and desired to report on (Tsujimoto et al., 2022). Knowing what to include and what to exclude in the search strategy is of utmost importance and following PRISMA reporting guidelines will help with transparency in the report.

When searching for evidence to include in an SR, the intention is to find the totality of literature using a global lens. To achieve this goal, researchers must look for relevant records from both the published and unpublished evidence, from full‐text articles to conference proceedings, and from author inquiries to other gray literature that would inform the review question. Finding the totality of evidence can be accomplished by searching multiple databases, looking at the references section of publications, and choosing large databases to search since it has been shown the size of the database will determine how comprehensive the search can be (Khiste et al., 2011). Plus, researchers must be cognizant that when searching general search engines such as Google, Yahoo, MSN, or Bing, the search engine results page (SERP) will be different since each search engine subscribes to different search engine optimization (SEO) protocols (Almukhtar et al., 2021). This is important because the behavior of information seekers is to view only the first two or three pages of a SERP. Knowing the SEO of a general search engine is an important aspect of finding the totality of evidence and reducing bias due to SERP (Gezici et al., 2021).

### Aims

There is a need to analyze the current state of author reporting practices in SRs. Guided by the PRISMA‐S extension, our study aimed to capture author patterns and trends in using databases, platforms, and study registries. Results from this study will inform the global community of the comprehensiveness of information sources used in SRs. Furthermore, we provide best practice recommendations when conducting SRs to reduce language bias, increase the number of data sources, and include a variety of data sources.

#### Research questions

In total, three questions were used to help describe the current state of author reporting practices when detailing data sources in the methods section of SRs. The first two questions provide general search patterns and practices. The last pertains specifically to the PRISMA‐S guideline on using study registries:

1. Which data sources were most often included in the methods section of systematic reviews?
2. What is the average number of data sources used in the methods section of systematic reviews?
3. What percentage of systematic reviews incorporated study registries as a data source?

## METHODS

### Design

This study employed an analytic cross‐sectional study design using a bibliographic analysis of data sources listed in the methods section of the individual SRs. Bibliographic design gathers information from published materials and employs statistics and quantitative analysis to describe the findings (Zupic & Čater, 2015). Therefore, the design used in our study is appropriate for the research questions. The results display frequencies based on major language categories that may raise awareness of coverage bias to countries, languages, or publishers.

### Sample

Using a nonprobability, convenience sampling method, data collectors searched for the sample of SRs published in 2019 to survey and document data sources listed in the methods section. The publication had to be labeled as a SR in the title to be included. In addition, the publication had to follow standardized SR methodologies such as PRISMA (Moher et al., 2009), Cochrane Collaboration (Henderson et al., 2010), JBI (Aromataris & Pearson, 2014), Campbell Collaboration (The Campbell Collaboration, 2017), or the National Academy of Medicine (formerly known as Institute of Medicine; Eden et al., 2011). The publication also had to specify data sources content in the Methods section. To provide robustness of the sample, the research team tested two different search platforms (PubMed and Web of Science) to gather the sample of SRs. The researchers chose the two platforms for the significant amount of health‐related information found, the search functionality, global representation, and the data collector's experience at searching for SRs using the EndNote x9 (Clarivate Analytics, PA, USA) data management system.

In addition, SRs from a variety of common health conditions were included. Exclusion criteria included publications other than those produced in 2019, or the title did not include “systematic review.” Language restriction was not specified, so it was neither an inclusion nor an exclusion criterion.

### Instrument

The research team created a standardized form and piloted the instrument on two SRs before data collection. Elements in the standardized form included instructions for data collectors on how to search in either platform (PubMed or Web of Science) via EndNote. Examples of search terms were given, and a screenshot was provided from a published SR of where to find the data sources in the methods section. The written instructions asked data collectors to write the authors' names, the publication title, the journal name (for future auditing purposes), and to go to the methods section of the article and list the data sources reported.

### Study procedure

The Institutional Review Board reviewed the protocol, determined that the study did not meet the definition for human subjects' research, and exempted the study. Therefore, the study was approved (IRB # 5190164) and the team proceeded with data collection.

A total of 107 data collectors were recruited to gather data from two cohorts over two non‐consecutive days in 2019. Data collectors had previously completed 10 h of training in applying search strategies, finding SRs, and using EndNote X9. Once they entered the room and gave their assent, they were systematically randomized to search in either the PubMed or Web of Science platform. They also received a card with the name of a common health condition identified by MedicineNet.com. Using the standardized instructions, data collectors used “title” as a search parameter and “year” as the delimiter to search for SRs. They chose two SRs by convenience from the list of results, retrieved the full‐text portable document format file, read the methods section, and listed all the reported data sources on the form. Data collectors retrieved information on 214 records.

### Auditing and cleaning data

To ensure accuracy during data entry, the researchers inspected each record together while entering the data into an Excel spreadsheet. If either researcher had questions about the record, the team accessed the reference, reviewed the full‐text article, and audited it immediately. After locating the methods section, the research team verified the information or deleted the records as needed. Twenty‐nine percent (*n* = 62) of the 214 records underwent a full audit due to: something listed as a data source that was not (e.g., PICO, PRISMA, PROSPERO, Meta‐analysis, randomized controlled trial, PAMORA); title of the article did not contain the word “systematic review” as instructed; only one source was listed in the data collection form, thus needing verification that the authors did use only one source; or the research team was unfamiliar with the listed data source. Of the 62 audited records, 47 records were either correct or needed some correcting, and 15 records were deleted. Reasons for deletion included: an inability to verify the record electronically (*n* = 3), the record was not a SR (*n* = 6), or the record was a duplicate (*n* = 6). The final sample size was *N* = 199 for analysis.

### Statistical analyses

Descriptive statistics were calculated from Excel spreadsheets to analyze frequency data using distribution, central tendency, and variability measures. More specifically, the research team summarized the results for the database language, computed the average number of data sources used per SR, and documented the percentage of SRs that included study registries. In addition, narrative notes expounded or explained details of the quantitative components.

## RESULTS

Key characteristics of data sources were analyzed to provide information on the sample characteristics and the three study questions. The tables depict patterns of the data sources and study registries that are the basis for author reporting practices when conducting searches for SRs.

### Sample characteristics

The 199 records exhibited data from four types of SRs: (a) effectiveness, (b) diagnosis, (c) prognosis, or (d) cost‐effectiveness. Despite randomization, there were slightly more SRs (*n* = 103, 52%) from PubMed, while the other half (*n* = 96, 48%) came from Web of Science. Seventy‐eight health conditions were represented in the SRs and came from medical, surgical, pediatric, obstetric, or mental health populations.

### Data sources included in systematic reviews

All the SRs reviewed were published in English, yet many data sources listed in the methods section came from non‐English‐language databases. The main language categories included in our sample were English, Chinese, Spanish, and Portuguese. Due to the similarities in root origins, syntax, high‐reading comprehension between the two languages, and practice of Latin researchers having access to both Spanish and Portuguese scientific literature, data from these two were grouped together. Results for each language data source are depicted in Tables 1, 2, 3.

**TABLE 1:** English Language Data Sources Most Often Included in Systematic Reviews in 2019

	PubMed *N* = 103 SRs	Web of Science *N* = 96 SRs	Total *N* = 199 SRs
Medline (PubMed) Medline (OVID) Medline (ProQuest)	102 (99%)	93 (97%)	195 (98%)
Embase (Elsevier)	69 (67%)	60 (63%)	129 (65%)
Cochrane Library Cochrane Database of Systematic Reviews (CDSRs) Cochrane Central Register of Controlled Trials (CCRCTs)	64 (62%)	48 (50%)	112 (56%)
Web of Science platform (Clarivate)	37 (36%)	29 (30%)	66 (33%)
Scopus (Elsevier)	21 (20%)	18 (19%)	39 (20%)
CINAHL or Academic Premier (EBSCOhost)	17 (17%)	11 (11%)	28 (14%)
Google Scholar	12 (12%)	13 (14%)	25 (13%)
PsychINFO (APA)	10 (10%)	7 (7%)	17 (9%)

**TABLE 2:** Chinese Language Databases Reported in Systematic Reviews in 2019

	PubMed *n* = 103 SRs	Web of Science *n* = 96 SRs	Total *N* = 199 SRs
China National Knowledge Infrastructure (CNKI)	12 (12%)	3 (3%)	15 (8%)
Wan Fang/Wang Fang (China Online Journals)	11 (11%)	2 (2%)	13 (7%)
Chinese Biomedical Literature (CBM)	9 (9%)	2 (2%)	11 (6%)
China Science and Technology Journal Database (CSTJ) VIP	8 (8%)	2 (2%)	10 (5%)
SINOMED	1 (1%)	0 (0%)	1 (0.5%)

**TABLE 3:** Spanish or Portuguese Language Databases Reported in Systematic Reviews in 2019

	PubMed *n* = 103 SRs	Web of Science *n* = 96 SRs	Total *N* = 199 SRs
Latin‐American and Caribbean Health Sciences Literature (LiLACS)	5 (5%)	3 (3%)	8 (4%)
Scientific Electronic Library Online (SciELO)	3 (3%)	1 (1%)	4 (2%)
Coordenação de Aperfeiçoamento de Pessoal de Nível Superior (CAPES) Brazilian Journal and Theses databases	0 (0%)	2 (2%)	2 (1%)
Biblioteca Virtual de Salud (BVS)	1 (1%)	1 (1%)	2 (1%)

Although there were over 60 English‐language resources listed, the top eight English language data sources documented in order of popularity were: Medline (98%), Embase (65%), Cochrane Library (56%), Web of Science (33%), Scopus (20%), CINAHL (14%), Google Scholar (13%), and PsycINFO (9%; Table 1). The overall percentage of English language data sources was comparable between the two platforms.

The use of Chinese language databases was the second‐largest category of data sources found in our sample of SRs (Table 2). The third category of data sources from languages other than English was Spanish or Portuguese (Table 3). However, the usage of non‐English‐language data sources comprised a small percentage. A caveat is that one SR listed the Chinese‐language SINOMED as a data source. SINOMED is a proprietary products company that only lists its own publications, demonstrating a potential conflict of bias. In the Spanish and Portuguese resources, one author mentioned using BIREME as a data source, but this is a misnomer. Neither platform nor database, BIREME is the Latin American and Caribbean Center on Health Sciences Information and provides technical support to the Virtual Health Library (BVS).

### Average number of data sources used in systematic reviews

The average number of data sources reported in a SR was *M* = 3.9 (SD 2), mode = 3, and ranged from 1 to 10 data sources per SR. Surprisingly, eighteen records (9%) of the sample used only one data source to complete their SR. SRs with less than 4 data sources was 50%. A significant finding is that there was a higher percentage of SRs in Web of Science using only one data source (Table 4).

**TABLE 4:** Number of Data Sources Used per Systematic Reviews in 2019

	PubMed platform *n* = 103 SRs	Web of Science platform *n* = 96 SRs	Combined Totals *N* = 199 SRs
1 data source	3 (3%)	15 (16%)	18 (9%)
2 data sources	18 (17%)	17 (18%)	35 (18%)
3 data sources	24 (23%)	22 (23%)	46 (23%)
4 data sources	18 (17%)	17 (18%)	35 (18%)
5 data sources	17 (17%)	12 (13%)	29 (15%)
6 data sources	8 (8%)	4 (4%)	12 (6%)
7 data sources	3 (3%)	5 (5%)	8 (4%)
8 data sources	6 (6%)	3 (3%)	9 (5%)
9 data sources	3 (3%)	1 (1%)	4 (2%)
10 data sources	3 (3%)	0 (0%)	3 (2%)

### Percentage of systematic reviews that included study registries

Of the 199 SRs investigated, 30% included study registries as data sources, with five percent more on the PubMed platform (Table 5). Most study registries were from the Cochrane Central Register of Controlled Trials (CCRCT) at 20%, while 6% were from Clinical Trials.gov. The rest of the registry data sources were from five specialized study registries. Since not all data sources carry the name “registry,” the research team visited the website of each data source to identify possible study registries.

**TABLE 5:** Percentage of Study Registries Listed as a Data Source in Systematic Reviews in 2019

	PubMed platform *n* = 103	Web of Science platform *n* = 96 SRs	Combined TOTALS *N* = 199 SRs
ClinicalTrials.gov	9 (9%)	3 (3%)	12 (6%)
Cochrane Central Register of Controlled Trials (CCRCTs)	19 (18%)	20 (21%)	39 (20%)
PROSPERO Registry for Systematic Review protocols	2 (2%)	1 (1%)	3 (1.5%)
WHO International Clinical Trials Registration Platform (ICTRP)	1 (1%)	1 (1%)	2 (1%)
National Health Service Central Register (NHSCR)	1 (1%)		1 (.5%)
International Association Cancer Registry (IACR)	1 (1%)		1 (.5%)
American College of Radiology Registry (ACR)		1 (1%)	1 (.5%)
TOTAL	33 (32%)	26 (27%)	59 (30%)

## DISCUSSION

Proper details and specifics about the search strategies used, comprehensiveness, and how data was gathered improves the reproducibility of an SR (Rethlefsen et al., 2021). Limiting to only one language, searching a few databases, or not including study registries will increase language, reporting, and publication biases and reduce the rigor of the SR. Our findings illustrate a need for improvement as only 8% or less of the included SRs used international data sources, 50% used one to three data sources, and only one‐third included registries. Conversely, over 52% of the SRs in our study listed four or more data sources in the methods section, which aligns with recommendations to use four data sources for SRs of effectiveness (Aagaard et al., 2016; Bramer et al., 2017).

The finding that Medline was the most frequently used data source (via PubMed, Ovid, or ProQuest) coincides with recently published data (de Kock et al., 2020; Opheim et al., 2019). As a result, the research team derived two critical points regarding using Medline as a data source. Medline can be accessed from either free or fee‐based platforms. If authors did not have access via the fee‐based, they could still access it using PubMed. Since almost 100% of the SRs used Medline, this can incentivize journal publishers to get their journals indexed in Medline.

The second most popular data source from our research was Embase (Elsevier) at two‐thirds. This finding is reassuring because Embase includes many journals PubMed does not. When researchers use these top two data sources to search for studies, the global coverage of potentially included studies increases tremendously. Favorably, our research showed that over half (56%) of the SRs investigated included Cochrane Library as a data source, either using the CDSR or Cochrane Central Registry for Clinical Trials, both of which specialize in providing synthesized research findings or clinical trials. Our results show there has been a significant shift from author reporting practices elucidated in the study by Bramer et al. (2017) when five years ago, the Cochrane Library was not one of the top four data sources used in SRs. We believe this change indicates a maturing understanding of and increased rigor implemented when authors construct SRs. It also points to an increased knowledge about Cochrane Library. This finding is encouraging but shows there is room for improvement.

Our results show that a small percentage (13%) of SRs use global sources (Chinese, Spanish, and Portuguese). Although other languages (e.g., French, German, and Russian) produce significant health sciences publications (Vera‐Baceta et al., 2019), it is important to note that our results did not support this previous finding, possibly because our sample of SRs came from PubMed and Web of Science, and not from Embase, which includes more European journals. Despite this caveat, our findings still show there is language bias in SRs found in PubMed and Web of Science.

Language bias and publication bias, which are clearly defined phenomena (Amano et al., 2016; Grzybowski & Kanclerz, 2019; Konno et al., 2020), reduce the dissemination and impact of knowledge globally and foster scientific egocentrism. These phenomena are part of the broader argument for the need to include study results from languages other than English in SRs. A recent position piece called for having heightened awareness of the harm language bias creates, and encouraged researchers to make extra efforts to reduce the bias by increasing collaboration with others who may have specific language skills that the team can utilize (Stern & Kleijnen, 2020).

A positive development is that non‐English‐language databases, like those identified in our study, offer an English search function and provide abstracts in both English and the host language. Plus, many journals will accept and publish English language manuscripts. This point is key to having researchers locate and access the global body of literature (Rasmussen & Montgomery, 2018). Given that search function, abstract, and reports are available in English, researchers should search more in non‐English‐language databases.

Our study identified seven study registries from either single, original clinical trials or for SRs. However, other valuable international registries should be considered when searching for global evidence. Clinical trial registries include the EU Clinical Trials Register, Chinese Clinical Trials Registry, Pan African Clinical Trials Registry, Brazilian Clinical Trials Registry, the US National Institutes of Health for Clinical Trial Registries, and the National Library of Medicine Registries of Randomized Control Trials.

The Systematic Review Data Repository by the Agency for Healthcare Research and Quality, JBI, and Campbell Collaboration registries, Open Science Framework, Figshare, and INPLASY are other global resources that could be used as data sources when searching. Using worldwide databases will provide the researcher with a more robust picture of the published research from a global perspective and can assist in being compliant to PRISMA‐S standards.

Results from this study provide additional details to keep in mind when conducting SRs. Moreover, when teaching the didactics on informatics and EBP, helping students learn best practice principles such as searching multiple databases, including study registries, and looking for databases in other languages will provide a breadth and range of findings and reduce bias in results. By becoming competent in these informatics skills, health care professionals can implement these sound techniques throughout their careers and continue to verify that their practice is based on evidence (Crabtree et al., 2017; Hanneke, 2018).

### Study limitations

Two methodological limitations were identified. Most often, authors only listed the Cochrane Library and did not specify using CDSR (database), CCRCT (study registry), or both. Since there was no way to parse out this information, the number of study registries used in SRs in 2019 may be higher. Second, although studies in this research project included SRs published in English from non‐English‐language journals from other countries, our results cannot be generalized to SRs published in other languages. The population of SRs in other languages may display different characteristics for the type of database, number of data sources included, and the use of study registries.

### Implications for future research

There are 16 items in the new PRISMA‐S guidelines. Areas for further research include tracking other sections of the PRISMA‐S guidelines to identify current practices and compliance with the guideline extensions. Managing records could also be explored in terms of deduplication. There are some items in the *information sources and methods* section of the PRISMA‐S guidelines that our team did not focus on in the current study. Detailing author practices in documenting database and platform as the complete data source or specifying which databases are searched simultaneously when using a platform should be articulated. Finally, valuable information about durability, popularity, and usability could be identified by continuing to track data sources globally. Answers to these research areas would contribute to data science for SR construction.

### Linking Evidence to Action

- Explore non‐English‐language databases to find abstracts, original research, and synthesized research in English.
- Use multiple data sources to ensure sufficient coverage to capture the body of scientific literature on a topic.
- Remember to search study registries, as they are a crucial resource for unpublished studies.
- Reduce potential biases through comprehensive searching practices.
- Follow PRISMA‐S guidelines when reporting data sources in manuscripts.

## CONCLUSIONS

Authors must be diligent when reporting data sources. The rigor of a SR includes being comprehensive, reproducible, unbiased, and valid. However, our results showed that half of the SRs in this study listed one to three data sources, risking comprehensiveness in capturing the global body of evidence. This article serves as a cautionary guide for authors who construct a SR. Adding study registries in the methods section as one of the information sources will produce higher quality SRs that adhere to PRISMA‐S extension guidelines.
